# The Role of COL5A2 in Patients With Muscle-Invasive Bladder Cancer: A Bioinformatics Analysis of Public Datasets Involving 787 Subjects and 29 Cell Lines

**DOI:** 10.3389/fonc.2018.00659

**Published:** 2019-01-15

**Authors:** Xiang-Yu Meng, Ming-Jun Shi, Zi-Hang Zeng, Chen Chen, Tong-Zu Liu, Qiu-Ji Wu, Shuo Li, Sheng Li

**Affiliations:** ^1^Department of Urology, Zhongnan Hospital of Wuhan University, Wuhan, China; ^2^Institut Curie, PSL Research University, CNRS, UMR 144, Paris, France; ^3^Department of Oncology, Zhongnan Hospital of Wuhan University, Wuhan, China; ^4^Department of Biological Repositories, Zhongnan Hospital of Wuhan University, Wuhan, China; ^5^Department of Laboratory Medicine, Clinical Laboratory Medicine and Center for Gene Diagnosis, Zhongnan Hospital of Wuhan University, Wuhan, China

**Keywords:** muscle-invasive bladder cancer, COL5A2, prognosis, IPD meta-analysis, bioinformatics

## Abstract

Bladder cancer (BC) is one of the most common malignancies. Two previous studies identified collagen type V alpha 2 (COL5A2) as a potential biomarker in BC, both are simple reanalysis of a single transcriptomic dataset without subgroup analysis for muscle-invasive BC (MIBC). We focused in MIBC patients and explored the role of COL5A2 from an integration perspective, using refined methodology covering individual participant data meta-analysis and bioinformatics analysis. Eight transcriptomic datasets of 787 MIBC patients (including one dataset containing genomic mutation information) and two drug sensitivity datasets of 29 cell lines in which more than 250 compounds were analyzed. We found subjects with increased COL5A2 gene expression exhibited poorer prognosis, and the power analysis confirmed adequate sample size. FGFR3 was the only gene differential mutated between the COL5A2 high and low expression groups. Differential expression and co-expression network analysis suggested potential association between COL5A2 expression and essential pathways involved in cancer invasion and dissemination, including cell adhesion, extracellular matrix organization, and epithelial-mesenchymal transition. Coordinately, analysis of drug screening datasets and gene-drug interaction also revealed COL5A2 expression linked to cell morphogenesis, angiogenesis, blood vessel development, and urogenital development. The utility and feasibility of COL5A2 for clinically applicable prognosis prediction and risk classification and the exact underlying molecular mechanism should be further investigated in subsequent studies.

## Introduction

Bladder cancer (BC) is one of the most common malignancies in terms of incidence worldwide; in China, BC is the 12th common cancer, and the most frequent urological malignancy (1, 2). In recent years, the incidence of BC has been increasing due to improved performance of detection strategies and population-aging (3). BCs are classified into two major distinct subgroups: non-muscle-invasive bladder cancer (NMIBC), and muscle-invasive bladder cancer (MIBC) (4, 5). NMIBCs often have a favorable prognosis following transurethral resection with or without intravesical chemotherapy or immunotherapy with Bacillus Calmette-Guérin (BCG). Currently, the treatment for MIBC basically relies on a multi-modal strategy combining surgical removal, radiation against the primary or metastatic lesions, and chemotherapy administered pre- or post-operation. Targeted therapy and immunotherapy may also be considered for patients with specific features (6, 7). However, regardless of continuous evolution of therapeutic strategies, the prognosis of MIBC patients remains poor, and knowledge of the molecular changes driving its carcinogenesis, progression, metastasis and resistance to treatment is still limited.

In the past decades, investigators have applied high through-put bioinformatic technology to cancer studies, in order to identify important biomarkers that may accelerate discovery and translation (8, 9). A number of the datasets are stored in repositories open to public. Researchers can make use of these datasets by reanalysis fit to their own study design or data mining from an integration perspective (10).

The gene COL5A2, i.e., collagen type V alpha 2 chain, encodes an alpha chain for one of the low abundance fibrillar collagens. Mutations in this gene are associated with Ehlers-Danlos syndrome, types I and II. In cancer study, a few reports have indicated its role in the pathological process in multiple cancers including the colorectal cancer and ovarian cancer (11, 12). Two previous studies identified COL5A2 as a potential biomarker in BC (13, 14). However, they were both simple reanalysis of a single dataset, used only transcriptomic data, and did not perform subgroup analysis for MIBC. Therefore, in the present study, we further explored the role of COL5A2 in MIBC patients from an integration perspective, using refined methodology.

## Materials and Methods

### Data Resources

Eight transcriptomic datasets involving 787 MIBC patients were included in this study, which were used to examine the prognostic significance of COL5A2 by individual participants data (IPD) meta-analysis (8–10, 15–20). The TCGA BLCA RNA-seq transcriptom dataset (*n* = 407) was used for functional enrichment analysis in terms of gene ontology (GO), Kyoto Encyclopedia of Genes and Genomes (KEGG) pathways and Broad Institute Hallmark 50 terms, and construction of co-expression network and subsequent analysis (10). The TCGA BLCA whole exome sequencing (WES) dataset (*n* = 407) was used for genomic mutation analysis (10). The COL5A2 expression and drug sensitivity IC50 data for 19 MIBC cell lines and 250 compounds were extracted from the Genomics of Drug Sensitivity in Cancer (GDSC) database (21), also from the Cancer Cell Line Encyclopedia (CCLE) database data for 26 MIBC cell lines and 24 compounds (22). The drug-gene interactions were parsed from the Drug Gene Interaction Database (DGIdb) (23). The basic information of the included data resources is shown in Table 1 and Supplementary Table 1.

**Table 1:** List of data sources.

**Identification**	N of MIBC units^*^	**Dimensions accessed**
TCGA BLCA	407	Expression profiling by sequencing, mutation profiling by sequencing, overall survival
CIT BLCA	73	COL5A2 expression by microarray, overall survival
E-TABM-147	22	COL5A2 expression by microarray, overall survival
GSE5287	30	COL5A2 expression by microarray, overall survival
GSE13507	62	COL5A2 expression by microarray, overall survival
GSE31684	78	COL5A2 expression by microarray, overall survival
GSE32894	51	COL5A2 expression by microarray, overall survival
GSE48276	64	COL5A2 expression by microarray, overall survival
CCLE	26	COL5A2 expression by sequencing, drug IC50
GDSC	19	COL5A2 expression by microarray, drug IC50
DGIdb	NA	Gene-drug interaction

* *Units can be subjects or cell lines. MIBC, muscle-invasive bladder cancer*.

### IPD Meta-Analysis

Before IPD meta-analysis concerning COL5A2's prognostic significance, the COL5A2 mRNA expression level was normalized into percentiles in each expression datasets. In brief, the actual rank of subjects according to the COL5A2 mRNA expression level was calculated. Then the actual rank was divided by the sample size and multiplied by 100 to obtain the percentile. The percentiles held information of relative COL5A2 mRNA expression across subjects, and could be used for interpretable across-dataset integration through a meta-analytic approach. The outcome of interest was overall survival (OS).

Eight transcriptomic datasets involving 787 MIBC patients were used to examine the prognostic significance of COL5A2 by IPD meta-analysis, which focused on two measures, i.e., the prognostic significance of grouping by COL5A2 (subjects were classified as high vs. low COL5A2 expression groups with a median cutoff) and of 10% relative increase in COL5A2 expression. We performed both one-step and two-step IPD meta-analysis. For one-step IPD meta-analysis, we used two different methods, i.e., the multilevel mixed-effects Cox proportional hazard (PH) model assigning random-effects to datasets to allow dataset-specific effects, and the robust-variance Cox PH model dealing the clustering of samples in datasets with a robust sandwich estimator. For two-step IPD meta-analysis, we first performed Cox PH analysis within each dataset, and then pooled the effect sizes, i.e., the hazard ratios (HRs). A fixed-effects model would be used if no significant heterogeneity across dataset was identified (*I*^2^ < 50% and Cochran Q Test's *P* > 0.1), otherwise a random-effects model would be used. Forest plots showing HRs and 95% confidence intervals (95%CIs) were generated to visualize two-step IPD meta-analysis results. Besides, we calculated power for one-step models to determine if adequate samples were included to support a confident conclusion. For the investigation of high vs. low expression groups, we performed log-rank tests and plotted survival curves for visualization. The formulas used for power calculation were listed below:

$$1\;-\text{ β }=\{\begin{array}{c} \Phi \left(-{\text{z}}_{1-\frac{\text{α}}{2}}+\sqrt{\text{np }\left(\text{1 - p}\right)\text{ φln }{\left(\text{HR}\right)}^{\text{2}}}\right),\text{for high vs}.\text{low }\\ \Phi \left(-{\text{z}}_{1-\frac{\text{α}}{2}}+\sqrt{{\text{nσ}}^{\text{2}}\text{φln }{\left(\text{HR}\right)}^{\text{2}}}\right),\text{ for }10\%\text{ increments} \end{array}$$

where α = 0.05 is the significance level, β is the Type II error, $z_{1-\frac{\alpha }{2}}$ is the 100(1 – α/2) percentile of the standard normal distribution N(0, 1), *Φ* is the cumulative distribution function of N(0, 1), p is the proportion of subjects in the high expression group, σ^2^ is the variance of normalized COL5A2 mRNA expression, n is the number of subjects, and *φ* is the probability of event (24).

### Differential Expression and Functional Enrichment

We divided subjects in the TCGA BLCA RNA-seq dataset into high vs. low COL5A2 expression groups with a median cutoff, and performed differential expression analysis between these two groups, using the limma/voom approach with empirical Bayes smoothing of gene-wise standard deviations and adjustment with false-discovery rate (FDR). The top 100 (smallest FDR) genes were included for functional enrichment analysis in terms of GO, KEGG pathways and Hallmark 50 terms, using hyper-geometric test.

### Mutation Analysis

We divided subjects in the TCGA BLCA WES dataset into high vs. low COL5A2 expression groups with a median cutoff, and identified genes with significantly different mutation pattern between the two groups, using chi-square test and FDR adjustment.

### Co-expression Network and Systems Biology Analysis

We used the TCGA BLCA RNA-seq dataset to construct co-expression network, using the weighted gene co-expression network analysis (WGCNA) approach (25). We extracted the module where COL5A2 was located and extracted this substructure from the overall co-expression network. We extracted the first principal component from the expression matrix of all nodes in this module, and calculated its prognostic significance using Cox model, as a measurement of the correlation between the module and OS. We performed functional enrichment analysis on nodes within this module. Then, we applied the HotNet2 algorithm to this module to examine if COL5A2 was within a subnetwork of greater importance in terms of survival (26). In brief, we calculated for each node in the WGCNA module containing COL5A2 the P value of Cox regression, and used its -log10 transformation as the initial node weight for the diffusion-oriented subnetwork identification algorithm. If COL5A2 was included in an identified subnetwork, we would apply functional enrichment analysis to this subnetwork.

### Drug Sensitivity Analysis

We extracted IC50 sensitivity data of compounds and COL5A2 mRNA expression for 29 MIBC cell lines (19 included in the GDSC database, 26 included in the CCLE database). We identified drugs whose sensitivity was moderately to highly associated with COL5A2 mRNA expression across MIBC cell lines, using Spearman correlation analysis. Drugs whose correlation coefficient's absolute value larger than 0.3 were considered, their target genes extracted from the DGIdb database, and analyzed concerning functional enrichment.

### Analysis Tools

The HotNet2 python commands were used to identify subnetworks. The WebGestalt application was used for functional enrichment (27). The remaining analyses implemented in this study were conducted using R 3.4.3 and the following packages: “survival,” “meta,” “coxme,” “limma,” “stats,” “WGCNA,” and “igraph.”

## Results

### IPD Meta-Analysis

**Figure 1:**
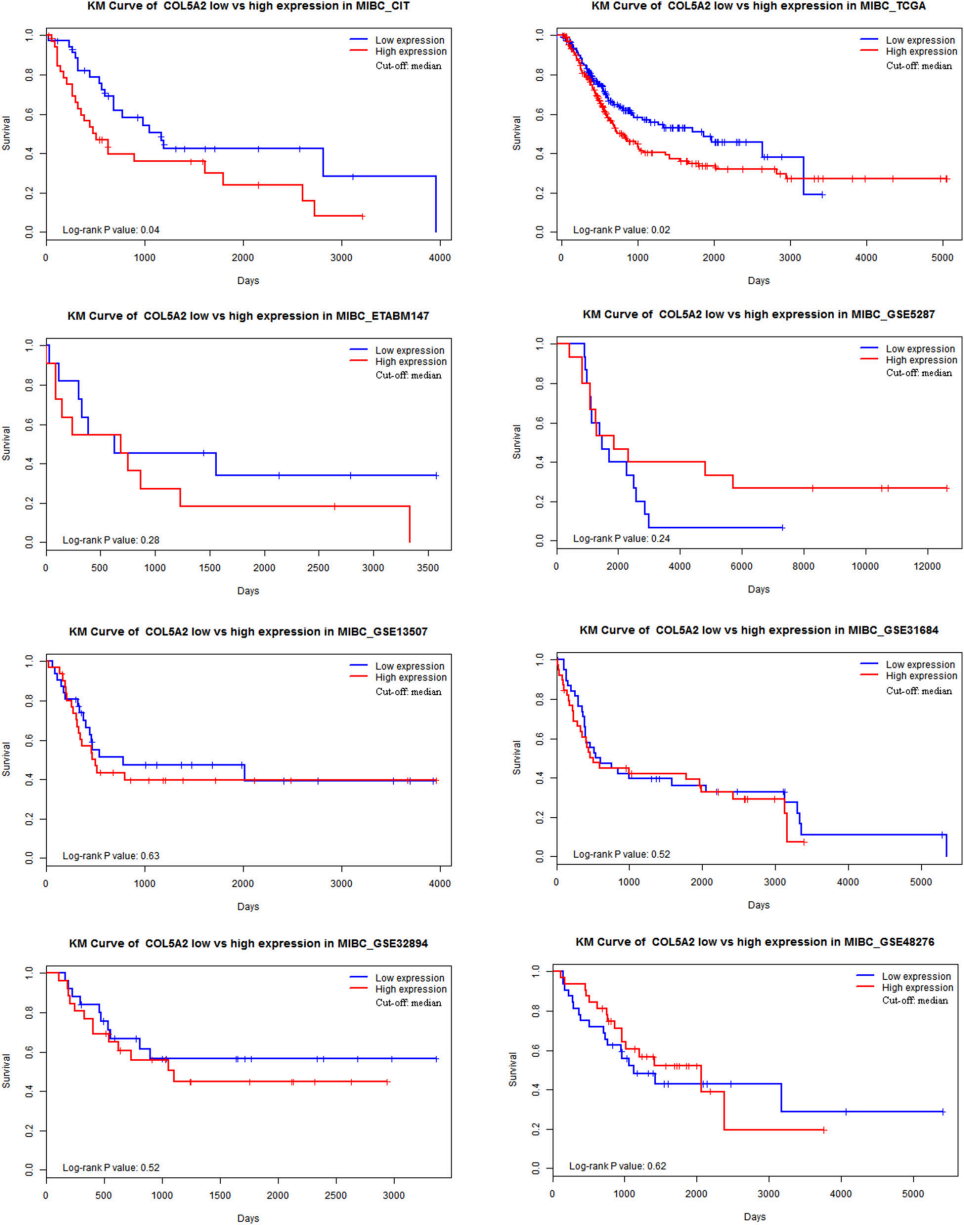
Kaplan-Meier curve of COL5A2 low vs. high expression in 8 datasets.

Eight transcriptomic datasets involving 787 MIBC patients were included to examine the prognostic significance of COL5A2 by IPD meta-analysis. The Kaplan-Meier curves for high vs. low expression in each dataset were shown in Figure 1. Significant association was observed in two datasets (TCGA and CIT), both indicating worse prognosis in COL5A2 high expression group. Non-significant association was observed in remaining datasets.

**Figure 2:**
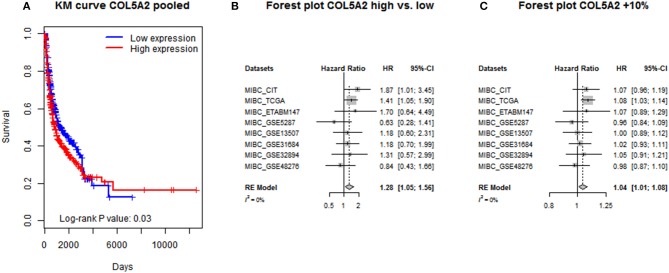
IPD meta-analysis of COL5A2's prognostic significance by **(A)** simple pooling; **(B)** two-step analysis for COL5A2 low vs. high; and **(C)** two-step analysis for COL5A2 10% increment.

From the results of one-step IPD meta-analysis using mixed-effects or robust variance Cox regression model, a significantly positive association was found between increased COL5A2 mRNA expression and poor prognosis measured by OS, in terms of both group comparison (Cox regression with mixed-effects: HR = 1.25, 95% CI = [1.03, 1.51]; Cox regression with robust variance: HR = 1.23, 95% CI = [1.05, 1.45]) and 10% increment (Cox regression with mixed-effects: HR = 1.04, 95% CI = [1.00, 1.08]; Cox regression with robust variance: HR = 1.04, 95% CI = [1.00, 1.08]). Regarding two-step IPD meta-analysis, no heterogeneity was detected among individual datasets (*I*^2^ = 0). Pooled effect sizes were very close to the ones calculated from one-step analyses (high vs. low expression: HR = 1.28, 95% CI = [1.05, 1.56]; 10% increment: HR = 1.04, 95% CI = [1.01, 1.08]). The Kaplan-Meier curve of merged data and forest plots of two-step IPD meta-analysis are shown in Figure 2.

As additional exploration, we also calculated power for one-step models to determine if adequate samples were included to support a solid conclusion. In terms of group comparisons, the power was 0.82 and 0.76 for mixed-effects and robust variance Cox models, respectively; for 10% increments, the power was 0.83 for both models. Therefore, acceptable Type II error and adequate power was achieved in all analyses.

### Differential Expression and Functional Enrichment

**Figure 3:**
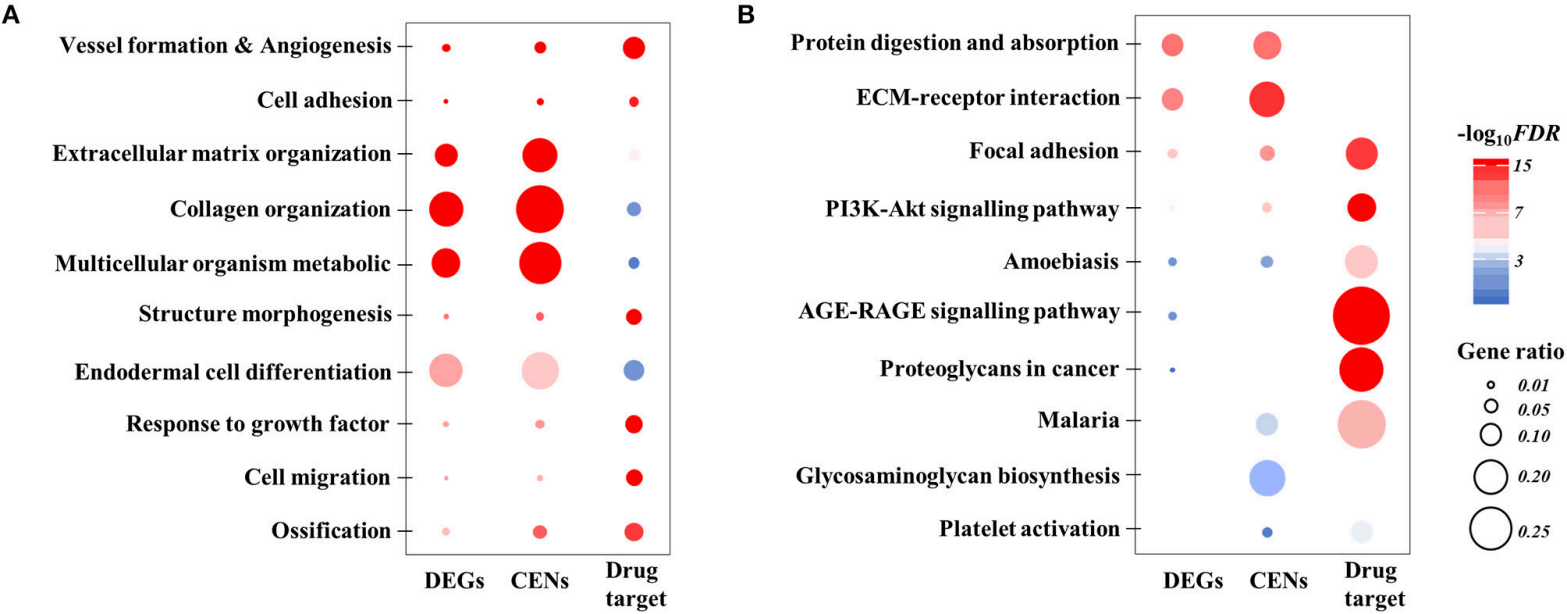
Visualized summary of functional enrichment analysis. **(A)** Top 10 gene ontology of biological process enriched with DEGs, CENs, and COL5A2-associated drug targets. **(B)** Top 10 KEGG pathways enriched with DEGs, CENs and COL5A2-associated drug targets. All with FDR < 0.05. The dot size represents gene ratio between our input list and total gene composition of a given term. The color scale represents the value of log10 FDR. DEGs, differentially expressed genes; CENs, co-expression networks; FDR, false discovery rate.

The top 100 differentially expressed genes are provided in Supplementary Material 1. The top 5 GO enrichment (biological process) terms were “blood vessel development,” “cell adhesion,” “biological adhesion,” “extracellular matrix organization,” and “collagen fibril organization” (Figure 3A; Supplementary Table 3). The top 5 KEGG enrichment terms were “protein digestion and absorption,” “ECM-receptor interaction,” “focal adhesion,” “PI3K-AKT signaling pathway,” and “amoebiasis” (Figure 3B; Supplementary Table 4). The top 5 Hall Mark 50 terms were “epithelial-mesenchymal transition (EMT),” “angiogenesis,” “coagulation,” “myogenesis,” and “apoptosis.”

### Mutation Analysis

The overall mutation load was almost equal between the two groups (47164 vs. 47006 mutations, respectively in the low and high expression group). The top 10 mutated genes in the COL5A2 low expression group (*n* = 204) were TTN (46.08%), TP53 (42.65%), KDM6A (30.88%), MUC16 (28.43%), KMT2D (26.96%), ARID1A (25%), FGFR3 (22.55%), KMT2C (22.06%), PIK3CA (20.10%), and RYR2 (19.12%). The top 10 mutated genes in the COL5A2 high expression group (*n* = 203) were TP53 (53.69%), TTN (48.28%), KMT2D (29.56%), MUC16 (28.57%), ARID1A (24.63%), PIK3CA (24.14%), SYNE1 (22.17%), and HMCN1 (21.18%). The top 50 differentially mutated genes are provided in Supplementary Table 5. The FGFR3 mutation status was the only one with significance as defined by FDR < 0.05.

### Mining of Co-expression Network

We constructed a co-expression network based on the TCGA BLCA transcriptomic dataset using WGCNA. K-means clustering was applied and genes were clustered into 22 modules. The COL5A2 genes were located within a module that contained 242 nodes (Supplementary Table 6). The first principal component (PC) of these nodes' expression matrix, which accounted for 54.46% of overall variance (the second and following PCs >4%), was significantly correlated with OS (Cox regression, beta = 0.02, *P* = 0.01). According to functional enrichment analysis of these node genes, the top 5 GO enrichment terms were “skeletal system development,” “blood vessel development,” “cell adhesion,” “biological adhesion,” and “extracellular matrix organization” (Figure 3A; Supplementary Table 3); the top 5 KEGG enrichment terms were “ECM-receptor interaction,” “protein digestion and absorption,” “focal adhesion,” “PI3K-Akt signaling pathway,” and “malaria” (Figure 3B; Supplementary Table 4); and the top 5 Hall Mark 50 terms were “epithelial-mesenchymal transition,” “coagulation,” “angiogenesis,” “apical junction,” and “myogenesis.”

In addition, we tested if the COL5A2 was one of the core nodes in its harboring module. Instead of applying traditional topological analysis, we performed the subnetwork mining with the HotNet2 approach, which could identify subsets of genes of importance in terms of both original correlation with phenotype and network topology. Very interestingly, as expected, the COL5A2 gene appeared in a subnetwork identified using HotNet2, which contained 20 node genes and 190 edges (Supplementary Figure 1).

### Drug Sensitivity Analysis

According to compounds sensitivity data and COL5A2 mRNA expression for 29 MIBC cell lines (19 included in the GDSC database, 26 included in the CCLE database) extracted, we identified 43 drugs' IC50 value in GDSC database and 5 drugs' IC50 value in CCLE were moderately to highly correlated with COL5A2 expression through spearman correlation analysis. As a result, there were a total of 48 drugs that showed sensitivity related to COL5A2 expression in MIBC cell lines.

Furthermore, we extracted the target genes of the 48 considered drugs from DGIdb database and performed functional enrichment in terms of GO and KEGG. A total of 349 unique target genes were identified. The top GO terms included “negative regulation of transcription from RNA polymerase II promoter,” “MAPK cascade,” “activation of MAPK activity,” “cell morphogenesis,” “cell morphogenesis involved in differentiation,” “angiogenesis,” “blood vessel development,” “urogenital system development,” and “ameboidal-type cell migration” (Figure 3A; Supplementary Table 3). The top KEGG terms included “endocrine resistance,” “ErbB signaling pathway,” “Rap1 signaling pathway,” “FoxO signaling pathway,” and “PI3K-Akt signaling pathway” (Figure 3B; Supplementary Table 4). The drugs, correlation coefficients between sensitivity and COL5A2 expression, and list of targeted genes are provided in Supplementary Tables 7, 8.

## Discussion

BC is a common cancer that affects the urinary tract. MIBC is a subclass of bladder cancer that presents high risk for poor prognosis. The treatment of MIBC generally requires radical cystectomy and aggressive adjuvant therapy; however, conventional treatment has limited effects in many MIBC patients, and brings risks of severe complications and adverse effects (3, 5–7). To this end, developing novel treatment targeting MIBC, especially those based on a personalized strategy is of great importance. For this, it is essential to identify biological markers that could be used to guide risk classification and to predict treatment response and prognosis.

A few reports have indicated COL5A2's role in the pathological process of cancers (11–14), including two studies that identified COL5A2 as a potential biomarker in BC. However, they are both reanalysis of a single dataset, used only transcriptomic data, and did not focus on MIBC. The MIBC and NMIBC manifest very different biological behaviors, thus analysis with mixed samples may result in inaccurate, misleading or biased findings. Therefore, in the present study, we further explored the role of COL5A2 in MIBC patients from an integration perspective, using refined methodology covering IPD meta-analysis and bioinformatics analysis. To the best of our knowledge, this is the very first study of its kind.

We conducted integration of multiple transcriptomic datasets by IPD meta-analysis, in order to examine the association between COL5A2 mRNA expression and MIBC patients' OS. We used two different parameters for comparison (high vs. low expression by median; and 10% expression increase) and performed both one- and two-step approaches of IPD meta-analysis as sensitivity analysis. Eight transcriptomic datasets and about 800 MIBC patients were included in the IPD meta-analysis. We showed subjects with increased COL5A2 mRNA expression have poor prognosis and a relatively 10% increase was associated with 4% increase of risk of poor survival on average. No significant heterogeneity was detected among the included studies. The results of one- and two-step analyses were quite similar. The power analysis confirmed adequate sample size included. Collectively, our study demonstrated that the COL5A2 mRNA expression was significantly correlated with OS, and can be considered as a candidate prognosis marker and for risk classification for MIBC patients.

In order to identify the underlying mechanisms and associated pathways of this gene-phenotype correlation, we performed different kinds of enrichment analysis. Either through GO enrich terms of biological process or by pathways enrichment within both differential expression analysis and co-expression networks, we found COL5A2 was highly correlated with cell extracellular matrix organization, vascularization and EMTs process which were known to be involved in cancer invasion and metastasis. This explains to some extent the observed prognosis predictiveness of COL5A2 expression. On the other hand, we also performed differential mutational analysis and identified that FGFR3 mutation was significantly enriched in the COL5A2 low expression group. Actually, it has been well-known that FGFR3 is one of the most frequently mutated genes in BC and is more frequent in lower tumor stage, with over 65% of NMIBC and about 15% of MIBC bearing an FGFR3 mutation (28, 29). We think this is the other way around to highlight the possibility that COL5A2 gene involved in much more malignant stages. However, the genomic driver of the aggressive tumors expressing more COL5A2 remains undetermined according to our results. As to the mutations with high frequency, taking TTN as an example, the observed high frequency may be linked to its length. Because this gene-level phenomenon solely depends on gene length and does not choose subject, the effects of this bias will be neutralized in differential mutation analysis.

In order to figure out potentially effective drugs targeting patients with high expression of COL52A, we evaluate the potential association between COL5A2 and drug sensitivity. We performed spearman correlation analysis considering the COL5A2 level and IC50 value of more than 250 screened compounds in 29 MIBC cell lines. We reported the major functions of these significantly associated compounds were collectively associated with cell morphogenesis, angiogenesis, urogenital development, cell migration and certain signaling pathways. These results are in line with the aforementioned enrichment observations, reinforcing the idea of COL5A2 being associated with tumor invasiveness and more malignant phenotype. However, the exact molecular mechanism behind COL5A2 still needs to be further investigated in subsequent studies.

To summarize, the present study with data mining by IPD-meta analysis, bioinformatics analysis and network analysis has demonstrated the prognostic predictive power of COL5A2 gene expression in MIBC patients. Based on a molecular glance, we speculated that COL5A2 expression might be closely linked to cancer invasion. For further investigation, the following issues should be addressed: (1) to explore the utility and feasibility of COL5A2 as a marker in clinical practice; (2) to verify the exact role of COL5A2 in cancer invasion, and elucidate whether it is a key player or just a bystander; (3) to further figure out in which pathways is COL5A2 really involved and to locate key mechanisms that may guide personalized treatment strategy.

## Author Contributions

X-YM and SheL designed the study. X-YM, M-JS, and Z-HZ conducted the analysis. X-YM and SheL drafted the manuscript. CC, T-ZL, Q-JW, and ShuL participated in result interpretation. All authors have read the manuscript and approved for publication.

### Conflict of Interest Statement

The authors declare that the research was conducted in the absence of any commercial or financial relationships that could be construed as a potential conflict of interest.

## Supplementary Material

Supplementary Figure 1 Subnetwork extracted by HotNet2 from the co-expression module containing COL5A2.

Supplementary Table 1 Basic information of included datasets.

Supplementary Table 2 Top 100 differentially expressed genes between COL5A2 high and low group.

Supplementary Table 3 Gene ontology of biological process enrichment among different datasets.

Supplementary Table 4 KEGG pathways enrichment among different datasets.

Supplementary Table 5 Differentially mutated genes between the COL5A2 high and low expression groups.

Supplementary Table 6 Node genes of the co-expression module containing COL5A2.

Supplementary Table 7 Correlation between COL5A2 expression and drug sensitivity.

Supplementary Table 8 Map of drugs (whose IC50 correlates with COL5A2) and target genes.
