# Supplementary figures and images for: The Role of COL5A2 in Patients With Muscle-Invasive Bladder Cancer: A Bioinformatics Analysis of Public Datasets Involving 787 Subjects and 29 Cell Lines
Source: Front Oncol. 2019 Jan 15;8:659. doi: 10.3389/fonc.2018.00659 (PMC6340941; doi:10.3389/fonc.2018.00659)

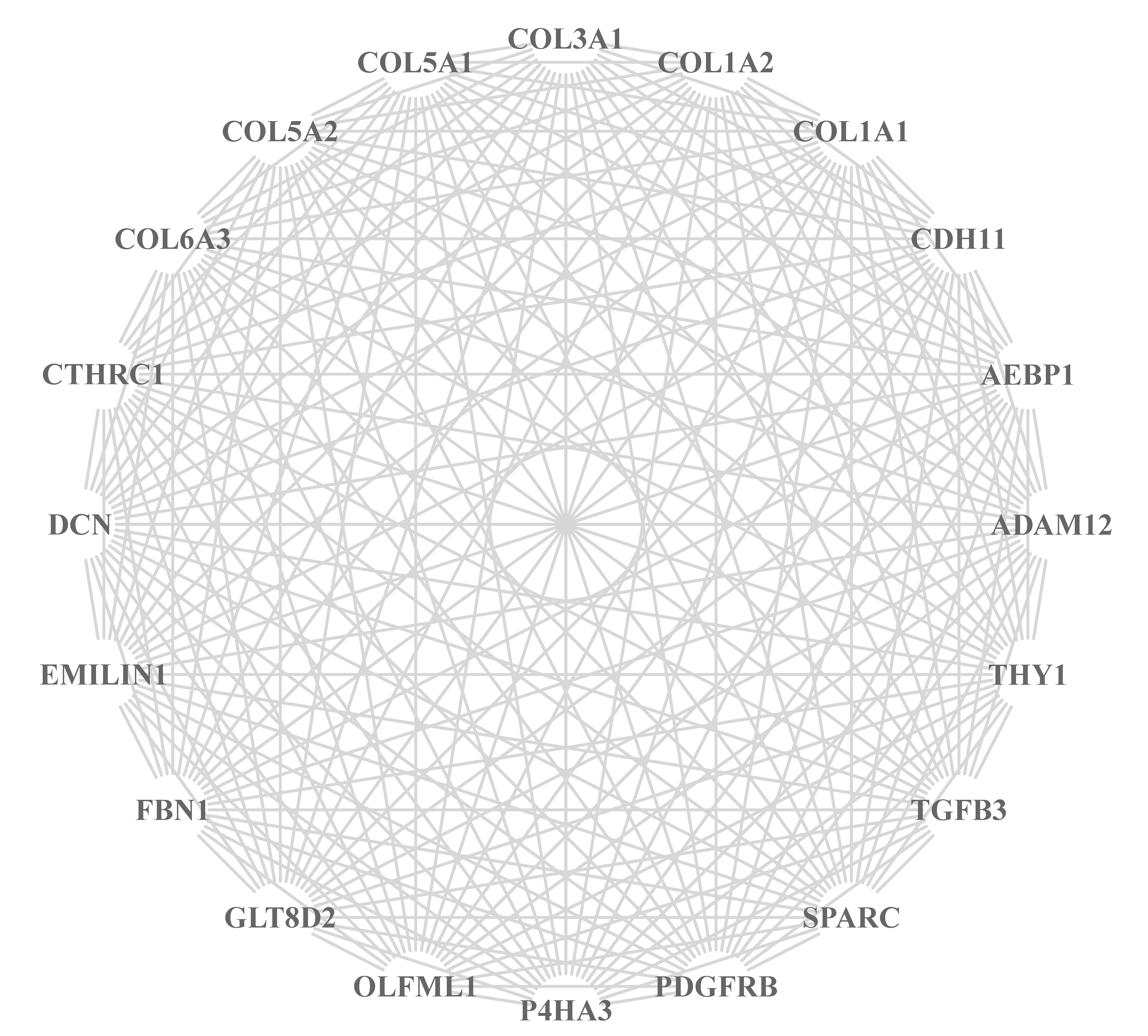

Supplement: Supplementary Figure 1 — Subnetwork extracted by HotNet2 from the co-expression module containing COL5A2. [file Image_1.tif]
